# Deformable elastic network refinement for low-resolution macromolecular crystallography

**DOI:** 10.1107/S1399004714016496

**Published:** 2014-08-29

**Authors:** Gunnar F. Schröder, Michael Levitt, Axel T. Brunger

**Affiliations:** a Institute of Complex Systems (ICS-6), Forschungszentrum Jülich, 52425 Jülich, Germany; b Physics Department, Heinrich-Heine University Düsseldorf, 20225 Düsseldorf, Germany; c Department of Structural Biology, Stanford University School of Medicine, Stanford, CA 94305, USA; d Howard Hughes Medical Institute and Departments of Molecular and Cellular Physiology, Neurology and Neurological Sciences, Structural Biology, and Photon Science, Stanford University School of Medicine, J. H. Clark Center, 318 Campus Drive, Stanford, CA 94305, USA

**Keywords:** deformable elastic network refinement, low resolution

## Abstract

Crystals of membrane proteins and protein complexes often diffract to low resolution owing to their intrinsic molecular flexibility, heterogeneity or the mosaic spread of micro-domains. At low resolution, the building and refinement of atomic models is a more challenging task. The deformable elastic network (DEN) refinement method developed previously has been instrumental in the determinion of several structures at low resolution. Here, DEN refinement is reviewed, recommendations for its optimal usage are provided and its limitations are discussed. Representative examples of the application of DEN refinement to challenging cases of refinement at low resolution are presented. These cases include soluble as well as membrane proteins determined at limiting resolutions ranging from 3 to 7 Å. Potential extensions of the DEN refinement technique and future perspectives for the interpretation of low-resolution crystal structures are also discussed.

## Abstract 2

An overview of applications of the deformable elastic network (DEN) refinement method is presented together with recommendations for its optimal usage.

## 1. Introduction   

Advances in sample preparation, data collection and analysis have enabled the structure determination of increasingly large systems such as protein complexes and membrane proteins by X-ray crystallography, such as the ribosome, transcription complexes and viruses (Schmeing & Rama­krishnan, 2009 ▶; Harrison, 2008 ▶; Kornberg, 2007 ▶). However, such challenging systems often display inherent flexibility or conformational heterogeneity, resulting in poorly diffracting and radiation-sensitive crystals. As a consequence, low-resolution data sets are commonplace for such systems (>3.5 Å). Advanced X-ray diffraction facilities such as undulator beamlines and hard X-ray free-electron lasers (XFELs) hold great promise to improve the limiting resolution by focusing on the better-ordered microdomains of a crystal. While it is likely that certain systems will continue to produce only low-resolution diffraction data even with these advanced light sources, the interpretation of such low-resolution data can still be of significant biological interest.

The interpretation of low-resolution diffraction data is generally difficult owing to the unfavorable ratio of parameters (variable degrees of freedom, such as flexible torsion angles or Cartesian atomic coordinates) to observables (observed diffraction intensities). From a purely numerical point of view, all dihedral angles of a protein should be fully determined at a resolution of 5 Å and with 50% solvent (the so-called determinacy point; Brunger, Adams *et al.*, 2012 ▶). A similar argument can be made for the determinacy point of nucleic acid structures or a mixture of both nucleic acids and proteins. Nevertheless, from a practical perspective we are far from reaching this goal. This is related to the need to interpret electron-density maps either manually (Emsley *et al.*, 2010 ▶; Jones *et al.*, 1991 ▶) or using automated methods (Terwilliger *et al.*, 2008 ▶; Langer *et al.*, 2008 ▶). Even at 3.5 Å resolution the interpretation of electron-density maps can be difficult, resulting in ambiguous models or, worse, errors in chain traces and side-chain positions. Furthermore, macromolecular refinement in reciprocal space can be problematic at resolutions worse than 4 Å and in the absence of high-resolution structures of the individual components of the system (DeLaBarre & Brunger, 2003 ▶; Davies *et al.*, 2008 ▶).

Although an exhaustive conformational search in torsion-angle space against the diffraction data should in principle produce an accurate structure at 5 Å resolution, such a search is at present computationally intractable. Thus, it is essential to aid the search by adding known structural information to the refinement target function at low resolution, in addition to generic information about macromolecular stereochemistry (the idealized chemical bond lengths, bond angles and atom sizes that heralded the era of reciprocal-space restrained refinement; Hendrickson, 1985 ▶; Jack & Levitt, 1978 ▶). The true structure of a macromolecule sometimes differs from a starting model (*e.g.* that obtained by homology modeling) by large-scale deformations, while the local geometry and packing are approximately conserved. An early approach (Diamond, 1990 ▶) used low-frequency normal modes, which were shown to reproduce large-scale collective changes in structures with very few degrees of freedom (Levitt *et al.*, 1985 ▶); this method has been used to refine protein structures with low-resolution X-ray or cryo-electron microscopy (cryo-EM) data (Delarue & Dumas, 2004 ▶; Tama *et al.*, 2004 ▶).

Deformable elastic network (DEN) refinement is a generalization of these early attempts to guide low-resolution refinement of structures against either X-ray or cryo-EM data (Schröder *et al.*, 2007 ▶, 2010 ▶). DEN refinement consists of torsion-angle refinement interspersed with *B*-factor refinement in the presence of a sparse set of distance restraints that are initially obtained from a reference model. The reference model can be simply the starting model for refinement, or it can be a homology model or even a predicted model that provides external structural information. In a typical application, the reference model is the search model used for molecular-replacement phasing. Thus, DEN refinement is a general method that only requires a starting model, making it similar to all other refinement methods. During the process of torsion-angle refinement with a slow-cooling simulated-annealing scheme, the DEN distance restraints are slowly deformed in order to fit the diffraction data. The magnitude of the deformation of the initial distance restraints is controlled by an adjustable parameter, γ, which is optimized by a global search for a minimum *R*_free_ value, possibly augmented by geometric validation criteria.

Here, we give an overview of DEN refinement. In the first part, we review the method and its strengths and limitations. In the second part, we present a representative set of controlled test cases and an actual example in which DEN refinement played a major role. Some of the examples have been previously published in detail, while others are reported here for the first time.

## 2. Description of the DEN method   

The DEN method was motivated by the observation that the refinement of macromolecules at resolutions worse than 4 Å often degrades the model instead of improving it, even when the starting model is a high-resolution crystal structure of the same macromolecule. Our design goal was to preserve the local structural information that was already present in the starting model, with automated inclusion of restraints during the refinement process. DEN refinement automatically detects which features in the model need to be changed in order to fit the diffraction data. This means that only those parts of the model are changed for which the diffraction data justify the change; all other parts are kept close to the starting model (the ‘null hypothesis’).

**Figure 1:**
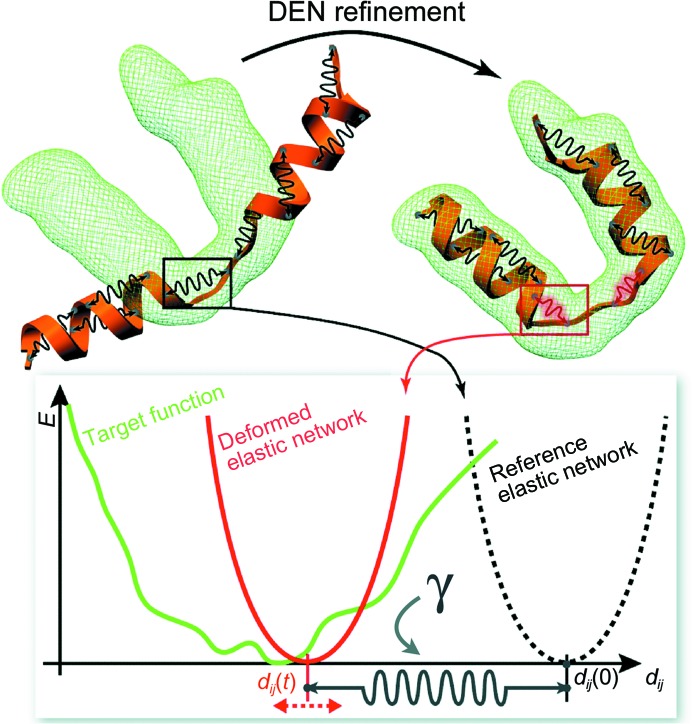
Illustration of the deformable elastic network (DEN) method. (*a*) A starting model (orange, left) is refined against experimental data such as a density map depicted by the green mesh. For DEN refinement, harmonic distance restraints (black springs) are defined between randomly chosen atom pairs in the starting model. During the refinement the equilibrium distances, *d_ij_*(*t*), of the restraints are allowed to change, as exemplified for one particular restraint in (*b*). The initial restraint potential (black dashed curve) is moved (red curve) in order to better fit to the electron-density map (green) (more generally, the target function *E*_target_; equation 2). The minimum of the DEN potential, *d_ij_*(*t*), is attached to the reference distance, *d_ij_*(0), by another harmonic potential, the strength of which is determined by the parameter γ. The choice of the parameter γ therefore reflects the relative influence of the reference model and the experimental data.

Fig. 1 ▶ illustrates the principle of the DEN method. A number *N* of distance restraints are defined between randomly chosen pairs of atoms that are within a specified distance range, typically between 3 and 15 Å, and that are separated in primary-sequence space within specified boundaries, typically not more than ten residues. For certain applications these default distances and sequence-separation limits should be modified, as will be discussed in the specific applications below. This list of atom pairs remains fixed during the particular DEN refinement, but they may be changed for ‘repeats’, where the refinement process is repeated with the same starting structure but with different random-number seeds for the initial velocity assignments and DEN atom-pair selections. The sum of these pairwise distance restraints is customarily referred to as an elastic network potential,
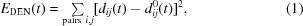
where *d_ij_*(*t*) is the distance between atoms *i* and *j* at time step *t* and *d* ^0^_*ij*_(*t*) is the corresponding equilibrium (target) distance of the restraint.

DEN refinement is by default performed using torsion-angle molecular dynamics (Rice & Brünger, 1994 ▶) with a standard crystallographic target function as implemented in *CNS* (Brunger, 2007 ▶; Brünger *et al.*, 1998 ▶) augmented by the *E*_DEN_ potential
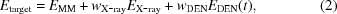
where *E*_MM_ is the Engh and Huber geometric force field (Engh & Huber, 1991 ▶). The term *E*_X-ray_ describes the deviation of the model structure factors from the measured structure factors, and *w*_X-ray_ and *w*_DEN_ are the weights of the corresponding energy terms. The refinement protocol typically uses a slow-cooling simulated-annealing scheme with a starting temperature of 3000 K cooling down to 0 K (the temperature is lowered in 50 K decrements; at each temperature level six steps of torsion-angle molecular dynamics are carried out with an integration time step of 4 fs, resulting in a total of 1.44 ps slow-cooling dynamics).

Positional refinement (*i.e.* *xyz* refinement) is usually combined with individual atomic *B*-factor refinement. However, since individual atomic *B* factors increase the number of refined variables, it may be more appropriate to use residue-grouped, restrained *B*-factor refinement, which means that typically two *B* factors (main chain and side chain) per residue are refined. At very low resolution, *B* factors of entire domains may be refined rather than residue-grouped *B*-factor refinement. The implementation of the DEN approach in *phenix.refine* (Adams *et al.*, 2010 ▶) also allows TLS refinement.

During a slow-cooling simulated-annealing scheme, the DEN equilibrium distances *d* ^0^_*ij*_(*t*) of these restraints are updated at each temperature-decrement step of the slow-cooling scheme using the equation
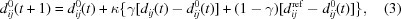
where *d*_*ij*_^ref^ is the distance between atoms *i* and *j* in the reference model. The right-hand side of (3) adds two terms to the current equilibrium distance *d* ^0^_*ij*_(*t*). The first term favors a shift of the DEN equilibrium restraints towards the current refined atomic coordinates, *i.e.* the restraints follow the motion of the model as it is being refined to fit the diffraction data. The second term favors an opposing shift of the DEN restraints towards the reference model (corresponding distances *d*_*ij*_^ref^). The parameter κ determines the speed at which the DEN restraints are changed. We typically use a value of 0.1, based on trial and error, in order to balance the overall speed of the refinement and allowing sufficient time for the conformational search during torsion-angle simulated annealing to take place.

The ‘deformation’ parameter γ is a value chosen between 0 and 1. It determines the degree to which the reference model distance information is kept during DEN refinement. Since the free variables (*i.e.* flexible torsion angles) are sampled by simulated-annealing molecular dynamics in order to fit the diffraction data, the γ value weights the influence of the reference model in the refinement process. For γ = 0 the DEN equilibrium restraints are fixed by the reference model. Consequently, no deformations of the DEN restraints are allowed. For a γ value between 0 and 1 only DEN equilibrium restraints that feel a large force from the *E*_X-ray_ term will be deformed, *i.e.* only those DEN equilibrium restraints for which the diffraction data provide significant information justifying the change. Other DEN equilibrium restraints will stay close to the reference model (depending on the γ value).

For the special case of γ = 1, the DEN equilibrium restraints track the motion of the model during the simulated-annealing process, albeit with some delay determined by the κ parameter. Therefore, the reference model is used more indirectly by providing an initial memory of the starting model that slowly dissipates during the simulated-annealing process. Hence, even with γ = 1 DEN guides the refinement process, but ultimately loses the memory of the initial DEN restraints from the reference or starting model. See below for a more in-depth discussion of this case.

We emphasize that DEN refinement does not use normal modes, and therefore DEN refinement is more general than methods that use flexible fitting guided by elastic normal modes (Tama *et al.*, 2004 ▶; Suhre *et al.*, 2006 ▶; Delarue & Dumas, 2004 ▶; Hinsen *et al.*, 2005 ▶, Tirion, 1996 ▶).

### 2.1. Parameter optimization   

The two most important parameters that need to be optimized for each individual refinement case are the γ value and the weight *w*_DEN_. Ideally, these parameters should be optimized by a grid search that consists of a large number of ‘trial’ refinements in order to find combinations of γ and *w*_DEN_ that yield low *R*_free_ values. The choice of which particular atom pairs are used for DEN restraints could also be optimized by additional trial refinements.

Although the DEN method does not require much manual intervention in that the network deforms itself where it needs to deform to fit the diffraction data, some additional improvement may be achievable by optimizing the DEN-restraints selection criteria. The upper cutoff for the distance range (which is typically set to 15 Å) can be decreased to allow more overall flexibility of the model or increased to include more structural information, especially at very low resolution. In addition to choosing the DEN restraints using a simple distance criterion, one can restrict the choice of atom pairs to atoms within a certain residue range along the peptide chain, typically 0–10 residues. These sequence-separation limits ensure higher flexibility between larger segments, for example to correct register shifts between helices while restraining local backbone and side-chain geometries. By default, no DEN restraints between different chains or, more generally, distinct molecules are used to give more freedom to the relative orientation between entire chains, which is typically well defined even by low-resolution data. However, there are cases where such inter-chain or inter-molecule restraints should be included, especially at very low resolution (see the photosystem 1 example discussed below).

### 2.2. Protocol   

By default, DEN refinement uses torsion-angle molecular-dynamics refinement with a simulated-annealing slow-cooling scheme against the target function *E*_target_ (2) as implemented in *CNS* (Brunger, 2007 ▶; Brünger *et al.*, 1998 ▶) and in *phenix.refine* (Afonine *et al.*, 2012 ▶). It is also possible to use Cartesian coordinate molecular-dynamics refinement or conjugate-gradient refinement against the target function *E*_target_, although the DEN restraints might then deform local geometry by ‘pulling’ on individual atoms. Moreover, performing Cartesian minimization may lead to overfitting at low resolution. Thus, *R*_free_ should be carefully monitored in order to decide whether a Cartesian minimization is warranted.

We recommend that 5–20 multiple repeats with different initial random velocities and random selections of DEN restraints be performed for each γ and w_DEN_ parameter pair. The results of the parameter grid search can be visualized by plotting *R*_free_ as a function of γ and w_DEN_. *R*_free_ contour plots often show a valley that tends to be diagonal, which means that the effect of decreasing the *w*_DEN_ value is often similar to increasing the γ value: both allow the model to deviate more from the starting or reference model. Nevertheless, the effect of decreasing the *w*_DEN_ value is not exactly the same as increasing the γ value: smaller *w*_DEN_ values weaken all restraints in the same way, while increasing the γ value changes those restraints more that are relevant to fit the diffraction data. It may be sufficient to perform a line search for the optimal γ value while keeping the *w*_DEN_ value constant (*e.g.* *w*_DEN_ = 100) or to sample *w*_DEN_ values on a coarser grid in order to reduce the computational cost of the grid search. Based on examining the results of grid searches for a variety of different crystal structures, we recommend a grid spacing of 0.2 for the γ value and an approximately logarithmic spacing for *w*_DEN_ (*e.g.* 3, 10, 30, 100, 300). Since the computational requirements for a full two-dimensional grid search are substantial, a grid-based computational resource is available through the SBGrid initiative (http://www.sbgrid.org).

If there is such a valley or band of low *R*_free_ values in the contour plot then it is unclear which DEN parameters are best; this is particularly true if the difference between the *R*_free_ values in the valley is not significant. We consider a difference between *R*_free_ values as significant if it is larger than two times the estimated standard deviation 1/*N* ^1/2^_test_, where *N*_test_ is the number of reflections in the test set. If the difference is not significant, we recommend the choice of the particular low *R*_free_ structure that has the best geometry, *i.e.* the best Ramachandran statistics, the smallest deviations from optimal bond lengths *etc*.

The equilibrium value of the DEN restraints is usually set to the starting coordinates, *i.e.* the model is at the minimum of *E*_DEN_ (1) at the beginning of the refinement in order to prevent large initial forces that could destabilize the model and lead to refinement artifacts. In the default protocol, we use nondeformable (γ = 0) restraints in the first macrocycle and then, in consecutive macrocycles, use an optimized γ value. This initial macrocycle relaxes the initial model and permits large structural rearrangements to occur before the local structure (such as side-chain conformations) is changed. When there is a large difference between the initial model and the reference model, it is also advisable to start with strong restraints, *i.e.* with a large *w*_DEN_ value.

We recommend testing whether refinement has converged to a stable local minimum of *E*_target_ by switching off the DEN restraints (*i.e.* *w*_DEN_ = 0) during the last two of the refinement macrocycles. If the model drifts significantly during these last macrocycles this could indicate that the model still contains substantial errors at limiting resolutions better than ∼4.5 Å. At lower resolution or when the diffraction data quality is poor, better results may be obtained by keeping the restraints active throughout.

If the model is already fairly close to the true structure, a single DEN refinement may suffice; in this case we recommend γ = 0 and *w*_DEN_ = 100. Even one pass of DEN refinement may produce an electron-density map that is superior to other types of refinement protocols since the method maintains perfect stereochemistry and thereby reduces the danger of overfitting.

The protocol as described here has been implemented in *CNS* (Brunger, 2007 ▶; Brünger *et al.*, 1998 ▶). An implementation of DEN refinement is also under development in *phenix.refine* (Adams *et al.*, 2010 ▶). Furthermore, the DEN method has been implemented in the real-space refinement program *DireX* (Schröder *et al.*, 2007 ▶; Wang & Schröder, 2012 ▶).

### 2.3. Effect of DEN restraints   

The effect of DEN restraints is to guide the refinement towards lower minima of the landscape of *E*_target_. Ideally, the DEN potential *E*_DEN_ does not ‘force’ the final refined structure but instead just provides a means to find the global minimum of refinement. As mentioned above, by default we therefore perform two macrocycles of torsion-angle simulated-annealing refinement without DEN restraints at the end of the process.

At a limiting resolution of 4 Å the target energy is expected to have a global minimum close to the true structure, and the *R*_free_ value is a good quantity to identify it. However, at limiting resolutions significantly lower than 4 Å additional restraints may be necessary in order to stabilize the refinement (Brunger, Adams *et al.*, 2012 ▶). 

DEN restraints guide the conformational search in torsion-angle space during simulated-annealing refinement against *E*_target_, thereby reducing the possibility of exploring physically unreasonable conformations. This is particularly useful for simulated-annealing refinement, where the initial high simulation temperatures could lead to movement into nonphysical regions of conformational space from which the model is unlikely to be able to move back closer to the true structure. In the presence of DEN restraints the model fluctuates around the DEN equilibrium distances and stays in the neighborhood of physically reasonable conformations. Since the minimum of *E*_DEN_ is updated as the model is refined (3), the DEN equilibrium distances move in a direction averaged over these local fluctuations. This effectively flattens the landscape of *E*_target_ and assists in moving towards the global energy minimum.

DEN restraints retain local information during refinement against low-resolution diffraction data and this limits the local conformational search. For example, side-chain and loop conformations will not be sampled as easily in comparison to regular simulated-annealing refinement. When the starting model contains significant errors, such as sequence mis­matches or incorrect loop conformations, such errors will generally not be corrected by DEN refinement and require complementary methods that operate in real space (Terwilliger *et al.*, 2008 ▶; Zwart *et al.*, 2008 ▶).

### 2.4. Reference model   

The reference model typically contains all of the structural knowledge that is initially available and that can be represented by a single modeled structure. Typically, this is the starting model for refinement, for example for phasing by molecular replacement the starting model will be the search model that was used for molecular replacement. The search model, in turn, can be a homology model based on one or more known high-resolution structures. Obviously, the closer the reference model is to the true structure, the greater the improvement that can be expected during refinement.

The reference model can in principle be different from the starting model. Often, a refinement is started from a preliminary model that was built into an electron-density map computed with either experimental phases or phases obtained by molecular replacement. As structures at higher resolution may become available, they could then be used as reference models in order to improve the current model. Nevertheless, we recommend that at subsequent stages of the refinement (*e.g.* during iterations of model building and refinement) the reference model be kept as the initial model (*e.g.* the molecular-replacement search model) and not updated with an already refined model.

Most improvement is expected when the reference model contains information that is truly complementary to the X-ray data. That said, the reference model does not have to be complete. It is possible to use DEN restraints for only parts of the model such as for single domains for which high-resolution structures are available. Several disconnected pieces are also allowed and these independent pieces could come from different sources, *e.g.* different crystal structures or a combination of crystal structures and homology models. These independent pieces do not need to be in a specific relative orientation if the distance selection criteria exclude inter-domain distances (this is the default setting).

### 2.5. Refinement with γ = 1   

In some cases, DEN refinement with γ = 1 can lead to improved models compared with using no DEN restraints at all. To explain this seemingly perplexing result, we refer to (3), which describes the updates of DEN restraints during refinement. For γ = 1, (3) becomes
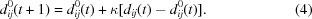



This update step is thus independent of the reference model; in particular, when the reference model is different from the starting model the reference model coordinates are never used. However, the equilibrium values of the DEN restraints are initially set to the starting model and then pulled into the direction of the atomic coordinates as they are being refined and fluctuate around the current DEN minimum. A particular DEN minimum will therefore move with the model coordinates along an averaged gradient, albeit with some delay as specified by the κ parameter. In this way, the memory of the starting model slowly dissipates over time, but it influences the trajectory of the refinement.

DEN refinement with γ = 1 effectively leads to a smoothing of the landscape of *E*_target_, which may improve the search for the global minimum of *E*_target_. We note that this smoothing of *E*_target_ does not affect the position of the local minima of *E*_target_: by definition, DEN refinement converges when a local minimum of *E*_target_ is reached. Convergence will be achieved when the DEN minima match the refined model. In this case, both the DEN potential and the forces on the atoms are zero, which means that the minima of *E*_target_ are the same as those of the target function in the absence of DEN restraints (*i.e.* with *w*_DEN_ = 0).

## 3. Comparison to other methods   

Other methods for assisted low-resolution crystallographic refinement can be viewed as special cases of DEN refinement or else are related to it. The local structural similarity restraints (LSSRs) in *BUSTER* (Smart *et al.*, 2012 ▶) penalize distance differences between the refined structure and a reference structure. The restrained atom pairs are typically chosen from a radius of 5.5 Å which includes local geometry and hydrogen-bonding residues. In contrast to DEN restraints, the LSSRs are not harmonic but have the form of a negative Gaussian function, such that the restraint forces are approximately harmonic only for small distance differences and approach zero for larger distance differences. This Gaussian function allows larger distance deviations without creating large forces. The DEN method is also able to handle large initial distance deviations by adjusting the minimum of the restraint potential; initially it is set to the value derived from the starting model and it is then slowly deformed towards the reference model. Similar external structure restraints can also be used in *REFMAC* (Murshudov *et al.*, 2011 ▶). In addition, *REFMAC* provides a regularizing method during the minimization of the target function, referred to as ‘jelly-body restraints’. A network of distance restraints is defined for atom pairs within a distance range (4.25 Å by default). The distances in the current structure serve as target values for the distance restraints. These restraints are however only used in the calculation of the second derivative during the minimization, *i.e.* they affect neither the target function nor the gradient, but instead change the search direction and so act as a regularizer during refinement. A related approach has been developed within the refinement program *phenix.refine* (Afonine *et al.*, 2012 ▶), referred to as ‘reference restraints’. These restraints are defined in torsion-angle space, where the target values are taken from the corresponding torsion angles in a reference structure (for example, a related structure determined to higher resolution). The form of the restraint potential is, as in the LSSRs in *BUSTER*, a negative Gaussian function.

Additional physical or statistical information can help to decrease the effective number of degrees of freedom of refinement, including restraints on hydrogen-bonding geometry (Fabiola *et al.*, 2002 ▶), Ramachandran-based backbone torsional potentials (Headd *et al.*, 2012 ▶) and electrostatics (Fenn *et al.*, 2011 ▶). Historically, the original implementation of crystallographic refinement by simulated annealing (Brünger *et al.*, 1987 ▶) used an early version of the CHARMM20 force field (Brooks *et al.*, 1983 ▶) that included electrostatics. The benefits of including electrostatics with respect to hydrogen bonding in crystallographic refinement were clearly noticed (Weis *et al.*, 1990 ▶), although some incorrect hydrogen bonds were observed when electrostatics were used during the simulated-annealing stages, especially for charged groups such as the head groups of arginine residues. As a result, it became the practice in subsequent protocols of simulated-annealing refinement to exclude electrostatics during all refinement stages (as was performed in other commonly used refinement programs) and assume that the diffraction data are capable of supplying this excluded *a priori* information (Adams *et al.*, 1997 ▶). However, in recent joint X-ray and neutron refinements, the hydrogen-bond orientation/geometry was improved by the inclusion of electrostatics in the force field during the final refinement cycles (Fenn *et al.*, 2011 ▶), suggesting that judicious inclusion of electrostatics in macromolecular structure refinement may be beneficial. Similarly, structure-prediction methods such as *Rosetta* (DiMaio *et al.*, 2013 ▶; Rohl *et al.*, 2004 ▶) that utilize potential functions developed for accurate structure recapitulation in the absence of diffraction data could be useful for crystallo­graphic refinement.

A variety of automated protein model-building tools are now available and, given sufficiently high-resolution data (3.0 Å or better) and reasonably accurate initial phase information, automated interpretation of electron-density maps is possible. However, with lower resolution data automatic interpretation generally fails, and manual building, when even possible, is difficult and prone to errors, which may be difficult to correct in refinement. Similarly, tools for auto-fitting coordinates into maps have been developed for RNA and DNA modeling, but suffer from similar problems when interpreting low-resolution data (Chou *et al.*, 2013 ▶).

## 4. Applications of DEN refinement   

In this section, we present five representative cases that illustrate the utility of DEN refinement for challenging refinement problems.

### 4.1. Large conformational changes during refinement   

Here, we demonstrate the potential of DEN refinement for cases where there are large conformational changes between the initial model and the true structure. The particular example is the β_2_-adrenergic receptor (β_2_AR), a membrane-bound protein consisting of seven transmembrane helices which belongs to the class of G-protein coupled receptors. The structure of its activated form was determined at a resolution of 3.5 Å (PDB entry 3p0g) in complex with an agonist and a nanobody that facilitated crystallization (Rasmussen *et al.*, 2011 ▶). The phases were originally determined by molecular replacement using the inactive β_2_AR structure (Cherezov *et al.*, 2007 ▶; PDB entry 2rh1) as a search model. Fully refining the structure required many rounds of ‘standard’ refinement and manual rebuilding.

We asked whether DEN refinement could have made the refinement process more efficient. We compared two refinement protocols: DEN refinement as implemented in *CNS* v.1.3 using default parameters and ‘standard’ refinement consisting of ten macrocycles with the *phenix.refine* program. Both protocols started from a molecular-replacement solution using the inactive β_2_AR structure as the search model and excluding the lysozyme that was present in the inactive β_2_AR structure (specifically, residues 29–230 and 263–342 of β_2_AR were included in the refinements). Moreover, the agonist (BI-167107) and the nanobody were not included in these test refinements. Molecular replacement was performed with *Phaser* (McCoy *et al.*, 2007 ▶) and yielded log-likelihood gain (LLG) and translation-function *Z* (TFZ) scores of 292 and 13, respectively.

**Figure 2:**
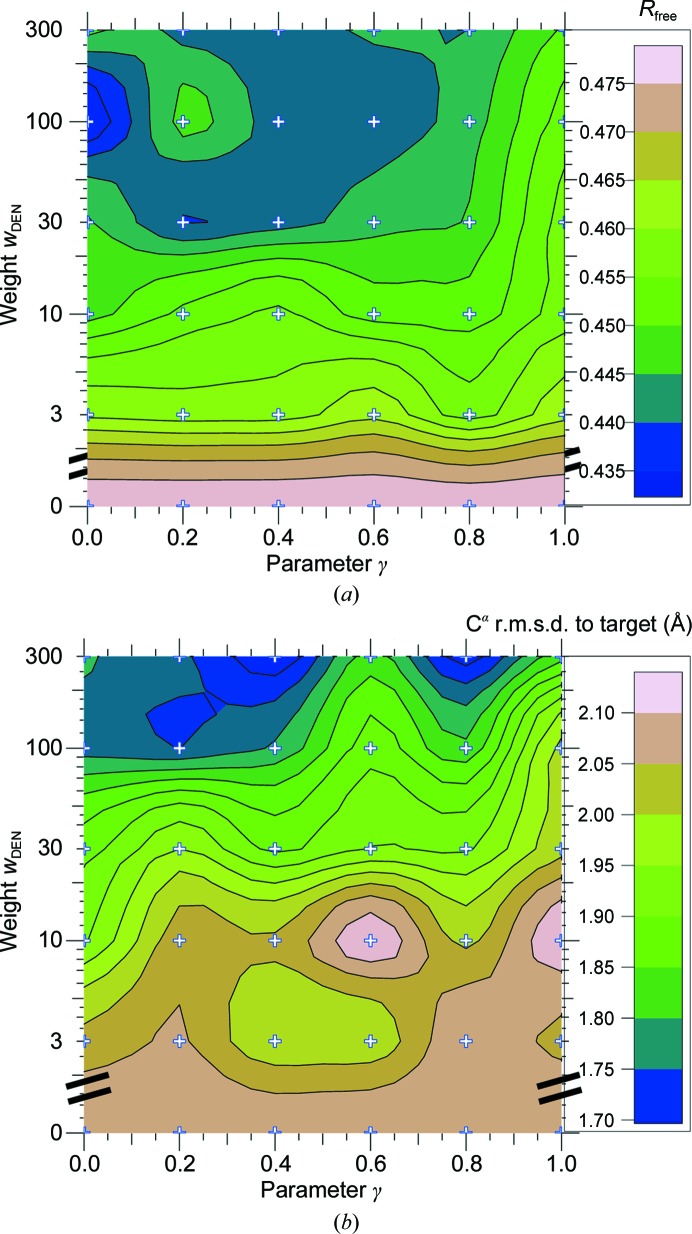
DEN parameter grid search for DEN refinement of the β_2_AR structure. (*a*) The *R*_free_ contour plot for combinations of the γ and *w*_DEN_ parameters. For each parameter pair, 20 refinement repeats were performed. (*b*) The corresponding contour plot for the C^α^ r.m.s.d. to the deposited structure (PDB entry 3p0g).

**Figure 3:**
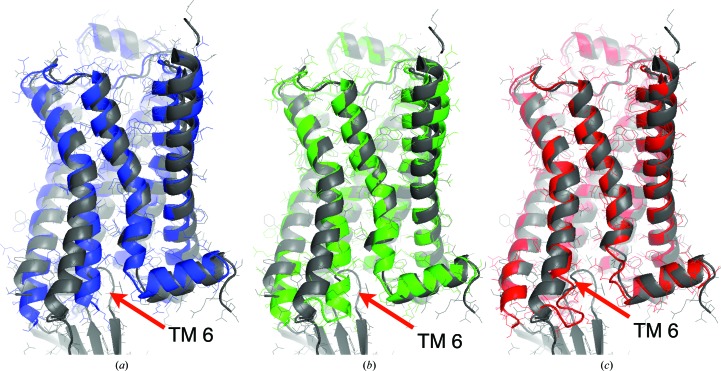
DEN refinement of the crystal structure of activated β_2_AR could have accelerated the process of iterative model building. The final deposited crystal structure of activated β_2_AR (gray; PDB entry 3p0g) is superimposed with (*a*) the crystal structure of the inactive form (blue; PDB entry 2rh1) that was used as a molecular-replacement search model, (*b*) a model (green) obtained by a ‘standard’ refinement procedure (ten macrocycles with *phenix.refine*) and (*c*) a model (red) obtained from DEN refinement. DEN refinement caused large movements towards the deposited structure (gray) in the transmembrane helices, in particular for helix TM 6 (red arrow). The C^α^ r.m.s.d. value between the DEN-refined model and the deposited structure (1.77 Å) is lower than that obtained by ‘standard’ refinement (2.09 Å; green model).

**Figure 4:**
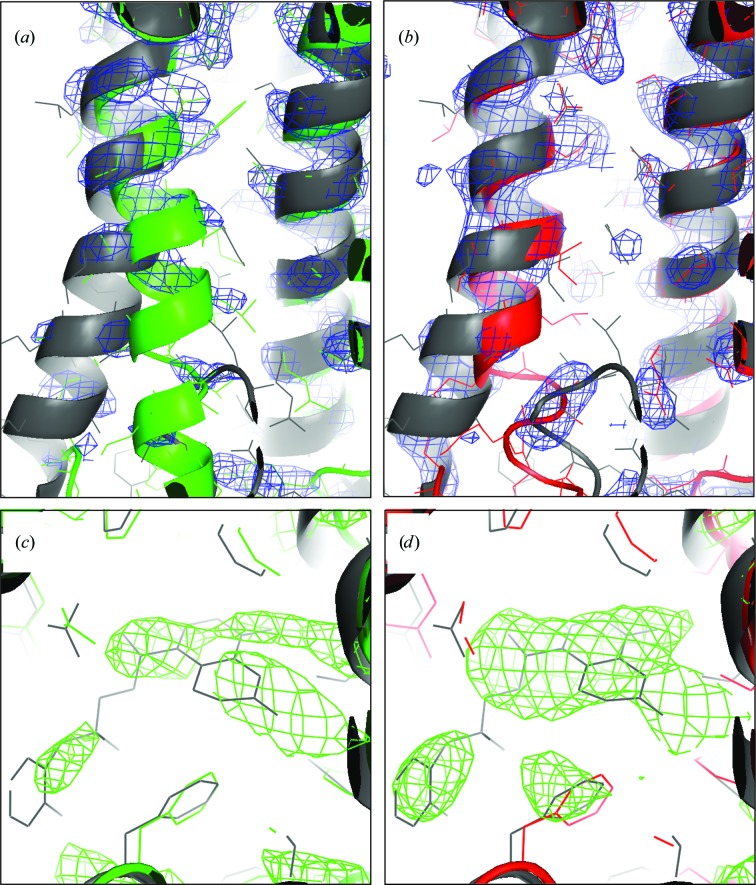
Electron-density maps of β_2_AR obtained with (*a*, *c*) ‘standard’ (green) and (*b*, *d*) DEN (red) refinement. The deposited structure (PDB entry 3p0g) is shown in gray. (*a*, *b*) Close-up view of helix TM6 superimposed on a 2*mF*_o_ − *DF*_c_ electron-density map at a contour level of 1.4σ (blue). DEN refinement (red) (*b*) leads to better resolved density: at least two more turns of the TM6 helix are visible compared with ‘standard’ refinement (green) (*a*). (*c*, *d*) A *mF*_o_ − *DF*_c_ difference density map (green) of the agonist (at contour level 2σ) is better defined for the DEN-refined model (*d*) than for the ‘standard’ refinement (*c*).

In Fig. 2 ▶, the parameter grid search (γ and *w*_DEN_) for the DEN refinement shows a good correlation of *R*_free_ with r.m.s.d. values to the deposited structure of the active form (PDB entry 3p0g). The parameter combination that yielded the lowest *R*_free_ value also produced a low r.m.s.d. value (only 0.07 Å higher than the best r.m.s.d. value of all trial refinements). DEN refinement withthe optimum parameter pair led to an overall better structure than ‘standard’ refinement and accomplished a larger part of the necessary structural changes. Fig. 3 ▶ shows the starting model (blue) and the structures obtained from DEN refinement (red) and from ‘standard’ refinement (green). We also show (gray) the comparison model that is the final refined structure as deposited in the PDB (PDB entry 3p0g). The overall r.m.s.d. value of the DEN-refined model is 1.77 Å, which is smaller than the value of 2.09 Å obtained with ‘standard’ refinement. The largest structural change accomplished by DEN refinement is a shift of transmembrane helix TM6 by 4 Å towards the true structure (*cf.* Fig. 3 ▶), whereas ‘standard’ refinement did not accomplish a significant improvement for TM6. In addition to the improved placement of TM6, the electron-density map obtained from DEN refinement shows clearly the last two TM6 helical turns (Fig. 4 ▶ *b*); the comparison electron-density map around TM6 obtained by ‘standard’ refinement is very poor (Fig. 4 ▶ *a*). The difference density maps for the agonist (Figs. 4 ▶ *c* and 4 ▶ *d*) also illustrate the higher quality of the DEN-refined model phases (Fig. 4 ▶ *d*).

### 4.2. Refinement at very low resolution   

Here, we illustrate that DEN refinement can produce more accurate models at resolutions that are close to the determinacy point; that is, the resolution at which the number of flexible degrees of freedom are comparable to the number of observed Bragg intensities in an asymmetric unit. The particular case that we studied (Brunger, Adams *et al.*, 2012 ▶) is photosystem I, a membrane-protein complex which consists of 2334 amino acids in 12 polypeptide chains. The original structure of PSI had been determined to a resolution of 2.5 Å (PDB entry 1jb0; Jordan *et al.*, 2001 ▶). Another low-resolution diffraction data set had been collected at the Advanced Light Source (ALS) at Lawrence Berkeley National Laboratory (LBL) at ∼6 Å resolution (Chapman *et al.*, 2011 ▶). For the refinement tests, we truncated the ALS diffraction data of photosystem I to 7.4 Å resolution in order to make them comparable to a data set collected with an X-ray free-electron laser light source [Linac Coherent Light Source (LCLS) at the SLAC National Accelerator Laboratory]. Refinements against the actual FEL data were not performed since these data suffered from an indexing ambiguity, resulting in a perfectly twinned data set and consequently significant model bias.

**Figure 5:**
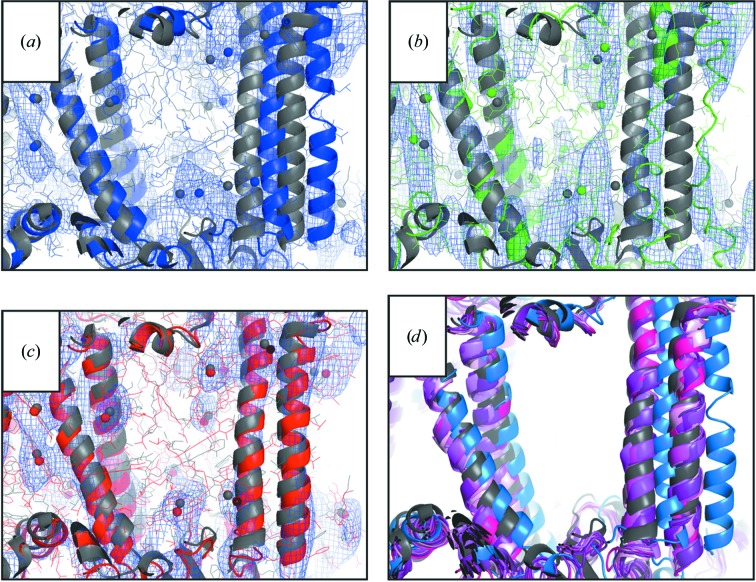
DEN refinement of photosystem I at 7.4 Å resolution illustrates that refinement at such low resolution is meaningful. (*a*) The crystal structure determined to 2.5 Å resolution (PDB entry 1jb0) is shown in gray. A perturbed starting model (blue) was used for the refinement tests. (*b*) Segmented rigid-body refinement followed by ‘standard’ refinement (200 steps of conjugate-gradient minimization against *E*_target_ with *w*_DEN_ = 0) yielded a structure that is relatively far away from the correct solution and that has poorly defined secondary structure. (*c*) Segmented rigid-body refinement followed by DEN refinement yielded a structure (red) that is very close to the high-resolution structure (PDB entry 1jb0). (*d*) The best ten models (in terms of *R*_free_) of refinement protocols that used segmented rigid-body refinement followed by DEN refinement from the two-dimensional grid search shown in Fig. 1 of Brunger, Adams *et al.* (2012 ▶). Blue, initial model. Gray, high-resolution crystal structure (PDB entry 1jb0). The best ten models are shown in different colors in the magenta/red range.

Starting models for the refinement tests were generated by perturbing the high-resolution crystal structure (PDB entry 1jb0) of photosystem I using unrestrained simulated-annealing molecular dynamics in torsion-angle space (Brunger, Adams *et al.*, 2012 ▶). One of the perturbed initial models had an r.m.s.d. of 4.3 Å to the high-resolution crystal structure (Fig. 5 ▶ *a*). All molecular-replacement and refinement tests used the truncated 7.4 Å resolution diffraction data of photosystem I (see above). Despite the large r.m.s.d., the perturbed initial model produced a molecular-replacement solution (Brunger, Adams *et al.*, 2012 ▶). The corresponding 2*F*_o_ − *F*_c_ electron-density map obtained with this starting model was of low quality and was not useful for model building (Fig. 5 ▶ *a*).

**Figure 6:**
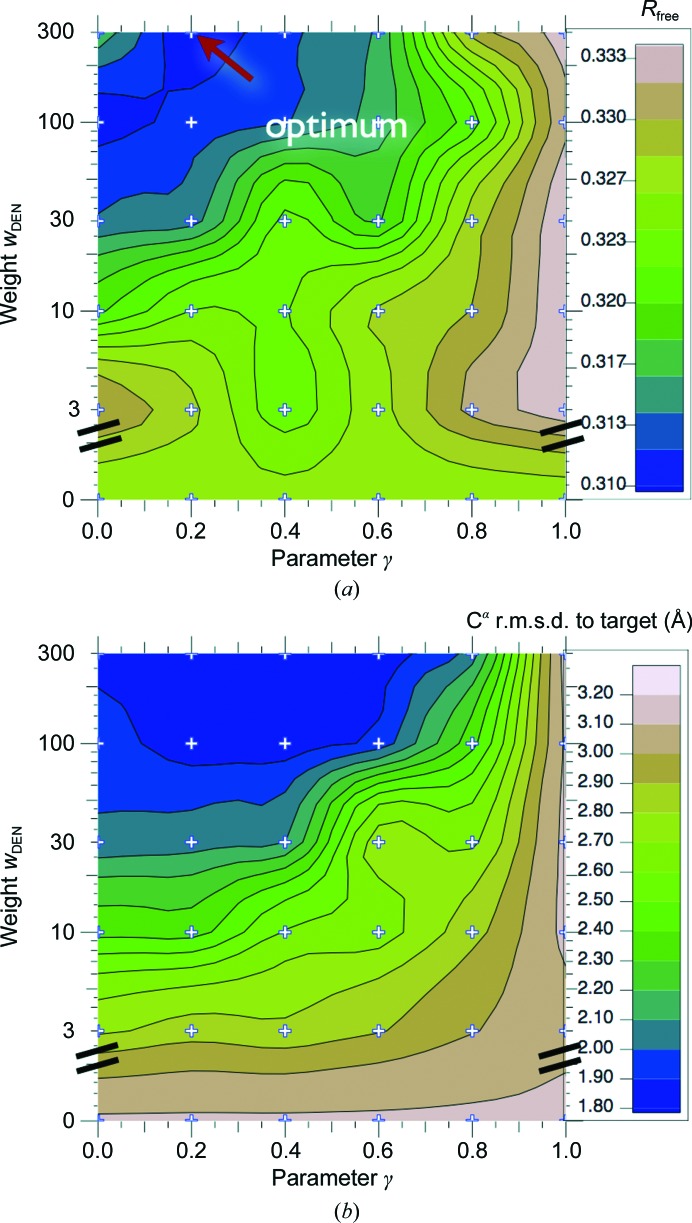
DEN parameter grid search for the re-refinement of full-length p97 in complex with ADP at 4.25 Å resolution. For each parameter combination 20 DEN refinement repeats were carried out. (*a*) The resulting *R*_free_ values are represented in a contour plot as a function of the γ and *w*_DEN_ parameters. (*b*) The r.m.s.d.s of the corresponding structures to the best available structure of p97 in complex with ADP (PDB entry 3cf3) are also shown as a contour plot. There is good agreement between the r.m.s.d. and *R*_free_ contour plots.

Initial tests showed that at such low resolution it is beneficial to first perform a segmented rigid-body refinement, *i.e.* to break up the model into pieces that move as rigid bodies. In the case of photosystem I, we first refined the 12 peptide chains and associated cofactors as individual rigid bodies before carrying out DEN or ‘standard’ (Brunger, Adams*, et al.*, 2012 ▶) refinement; this initial ‘segmented’ rigid-body refinement significantly improved the refined models although DEN refinement performed well even without such ‘pre’-refinement (Brunger, Adams *et al.*, 2012 ▶). The DEN refinement was performed with DEN restraints restricted to between atom pairs separated by 3–15 Å in the initial model (the default distance range). However, in contrast to the default DEN refinement protocol, no sequence separation limit was used, so that restraints were also present between the 12 peptide chains and all cofactors, thereby restraining their relative positions and orientations. By default, the starting model was also used as the DEN reference model and default values were used for all other DEN refinement parameters. A global search was performed (Fig. 6 ▶ *a*) giving good correlation between the *R*_free_ value and the r.m.s.d. to the high-resolution crystal structure (Fig. 6 ▶), which is taken as a measure of the correctness of the model. This demonstrates that cross-validation using the *R*_free_ value works well even at a very low resolution of only 7.4 Å. The best DEN refinement yielded *R*_cryst_ and *R*_free_ values of 0.29 and 0.38, respectively.

The comparison ‘standard’ refinement consisted of 200 steps of conjugate-gradient minimization with *CNS* without DEN restraints. The DEN-refined model was closer to the high-resolution crystal structure than to the structure obtained by ‘standard’ refinement (compare Figs. 5 ▶ *c* and 5 ▶ *b*). Specifically, with DEN refinement 60% of the atoms had r.m.s. deviations of less than 2 Å from the high-resolution crystal structure, compared with just 12% with ‘standard’ refinement. The r.m.s.d. of the DEN-refined structure to the high-resolution crystal structure was 2.4 Å compared with 3.5 Å for ‘standard’ refinement. Note that both refinements started from the segmented rigid-body refined model. Accordingly, the quality of the electron-density maps obtained from the DEN-refined structure (Fig. 5 ▶ *c*) was significantly higher than those obtained by ‘standard’ refinement. In fact, ‘standard’ refinement produced a biased electron-density map that showed spurious electron density for the two helices on the right in Fig. 5 ▶(*b*).

The extent to which independently refined models ‘converge’ is related to the power of the refinement method and to the quality (or information content) of the diffraction data. The large number of repeat refinements during the DEN grid search allows one to assess the convergence of refinements that equally well match the diffraction data. Fig. 5 ▶(*d*) shows the ten best models (in terms of *R*_free_) obtained by the grid search shown in Fig. 1 of Brunger, Adams *et al.* (2012 ▶). Note that each of the individual ten refinements used different randomly selected DEN restraints and initial random-number seeds for the starting velocities of the simulated-annealing molecular-dynamics refinements. Re­markably, these ten refinements all converged to a similar conformation close to that of the high-resolution crystal structure, suggesting that the convergence of DEN refinements is quite robust and that this particular set of diffraction data determines a unique conformation for well defined secondary-structural elements.

### 4.3. Re-refinement of a model with errors using an improved reference model   

Here, we illustrate that DEN refinement can correct a model that contains significant errors if a better reference model has become available after the initial model had been constructed. The particular test case is AAA-ATPase p97, a hexameric protein complex in which each of the protomers contains an N-terminal domain and two nucleotide-binding domains: D1 and D2. The two nucleotide-binding domains have a sequence identity of 40%. Structures were originally obtained for several nucleotide states of the hexameric complex of full-length p97 (DeLaBarre & Brunger, 2003 ▶, 2005 ▶).

The original structure of the ADP-bound complex (PDB entry 1yqi; DeLaBarre & Brunger, 2003 ▶) was determined at a resolution of 4.25 Å using a combination of multiwavelength anomalous dispersion (MAD) phasing and molecular replacement. The molecular-replacement search model consisted of the known structure of the N-D1 fragment of p97 that had been determined previously at 2.9 Å resolution (Zhang *et al.*, 2000 ▶). Since the structure of the D2 domain was unknown at the time, the D2 nucleotide-binding domain was modeled based on the structure of the D1 domain (DeLaBarre & Brunger, 2003 ▶, 2005 ▶). Performing iterations of manual inspection of electron-density maps interspersed with refinement resulted in relatively poor secondary-structure definition and several ‘register shifts’ (Brunger *et al.*, 2009 ▶).

When the crystal structure of the isolated D2 domain became available at 3 Å resolution several years later (Davies *et al.*, 2008 ▶), a new composite starting model comprising the high-resolution structures of the individual N, D1 and D2 domains was refined against the low-resolution diffraction data of p97 in three nucleotide states (Davies *et al.*, 2008 ▶). This re-refinement led to dramatic improvements compared with the original structures for *R*_free_, *R* − *R*_free_, secondary-structure geometry and fit to phase-combined electron-density maps, especially for the D2 domain. These improvements suggest that low-resolution diffraction data contain the information needed to assess the quality of a refined model and therefore indicate whether a particular model is significantly better.

**Figure 7:**
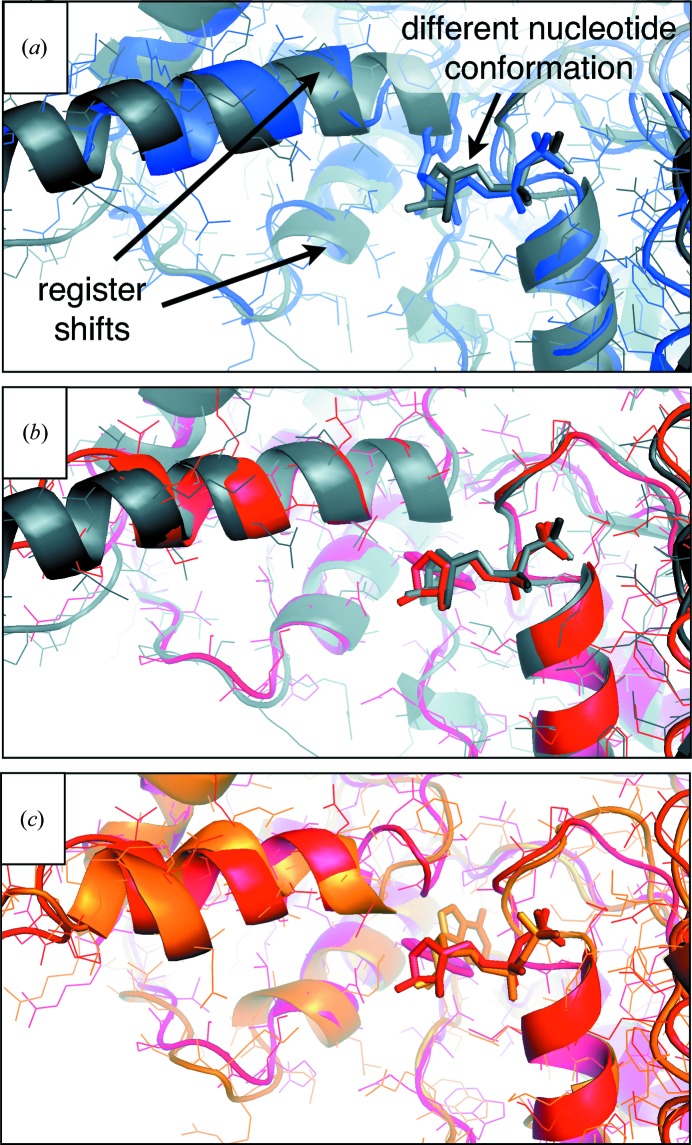
The D2 domain of full-length p97 posed a challenge for ‘standard’ refinement prior to the availability of a high-resolution structure of D2. We show a close-up view of the region around the nucleotide-binding site of D2 in complex with ADP. (*a*) The best available structure of p97 in this nucleotide state (gray; PDB entry 3cf3) is superimposed on the original structure (PDB entry 1yqi), which was determined without knowledge of the D2 domain of the crystal structure (unavailable at the time). This original structure had severe problems, *e.g.* a wrong orientation of the ADP molecule and some register shifts in helices close to the nucleotide-binding site in the D2 domain (black arrows). (*b*) The original structure (PDB entry 1yqi) was re-refined with DEN as described in the text. It yielded a considerably improved structure (red), with corrected register shifts and position of the nucleotide. (*c*) ‘Standard’ refinement (orange; see text) did not move the model closer to the true structure and could not correct the position of the nucleotide. Loops and α-helices show significant differences from the DEN-refined structure (red).

The original p97 structures used ‘standard’ refinement techniques which were unable to improve the quality of the models. Therefore, we chose to determine whether, in retrospect, DEN refinement could have helped to improve the original model that contained the errors and register shifts (see above), but without using the high-resolution structure of the isolated D2 domain. To this end, we re-refined the deposited original structure in the ADP nucleotide state (PDB entry 1yqi; Fig. 7 ▶ *a*, blue). A reference model was constructed using the higher resolution crystal structure of the N and D1 domains (Zhang *et al.*, 2000 ▶) and a homology model of the D2 domain. The homology model of D1 was obtained using *MODELLER* (Sali & Blundell, 1993 ▶) based on the structure of the D1 domain (from PDB entry 1e32). It turns out that the homology model of D2 had an r.m.s.d. of 3.1 Å to the crystal structure of the isolated D2 domain (PDB entry 3cf0). This reference model was of sufficient quality to produce a molecular-replacement solution for the p97 data set.

For DEN refinement, an optimal parameter pair (γ, *w*_DEN_) was obtained by a two-dimensional grid search (Fig. 6 ▶ *a*). We compared the DEN-refined models with the re-refined structure of p97 in the ADP nucleotide state (PDB entry 3cf3; Fig. 7 ▶, gray cartoon; Davies *et al.*, 2008 ▶). For comparison, refinement was repeated without DEN restraints. Fig. 7 ▶ illustrates that DEN refinement (red model) partially corrected a register shift present in the starting model (blue), while the refinement without DEN restraints actually led to a worse structure. Remarkably, the initially incorrect conformation of the ADP nucleotide was corrected by DEN refinement (Fig. 7 ▶ *b*), but not without DEN (Fig. 7 ▶ *c*). Thus, this example shows that it may have been possible to obtain a good model for the structure of the p97 complex with ADP by using DEN refinement without knowledge of the high-resolution crystal structure of the D2 domain.

### 4.4. Strong phase improvement   

Here, we describe how DEN refinement played an essential role in the structure determination of a low-resolution crystal structure, that of the human myxovirus resistance protein (MxA), a dynamin-like GTPase which acts as a host restriction factor against many viral pathogens (Gao *et al.*, 2011 ▶). The crystals diffracted anisotropically to a limiting resolution of 3.5 Å. The crystal structure of MxA consists of three domains: the nucleotide-binding G domain, the bundle signaling element (BSE) and the stalk, which is a four-helix bundle. MxA forms higher-order filamentous and ring-shaped oligomers, in which the subunits assemble *via* the stalk domain and are further stabilized by interaction of the BSE domain with the stalk domain of the neighboring subunit.

The structure of MxA was determined by molecular-replacement phasing with *Phaser* (McCoy *et al.*, 2007 ▶) using the previously determined structures of the MxA stalk (Gao *et al.*, 2010 ▶) and the nucleotide-free G domain of the homologous dynamin (Reubold *et al.*, 2005 ▶) as search models. The homology model for the G domain was built based on the nucleotide-free rat G domain of dynamin using *SWISS-MODEL* (Arnold *et al.*, 2006 ▶).

After molecular replacement, the electron density for the G domain was very fragmented and poorly defined. However, electron density for the BSE domain was clearly visible and a model could be constructed with *Coot* (Emsley & Cowtan, 2004 ▶; Emsley *et al.*, 2010 ▶). The resulting complete model (*i.e.* the MxA stalk domain, the BSE domain and the G domain) was subjected to DEN refinement with *CNS*. The previously known crystal structures of the G domain and the stalk were used as the reference model for the DEN restraints, which contained 87% of all protein atoms. To verify the sequence assignment, the positions of nine methionines were determined by calculating an anomalous difference Fourier map from selenomethione (SeMet)-substituted MxA crystals.

**Figure 8:**
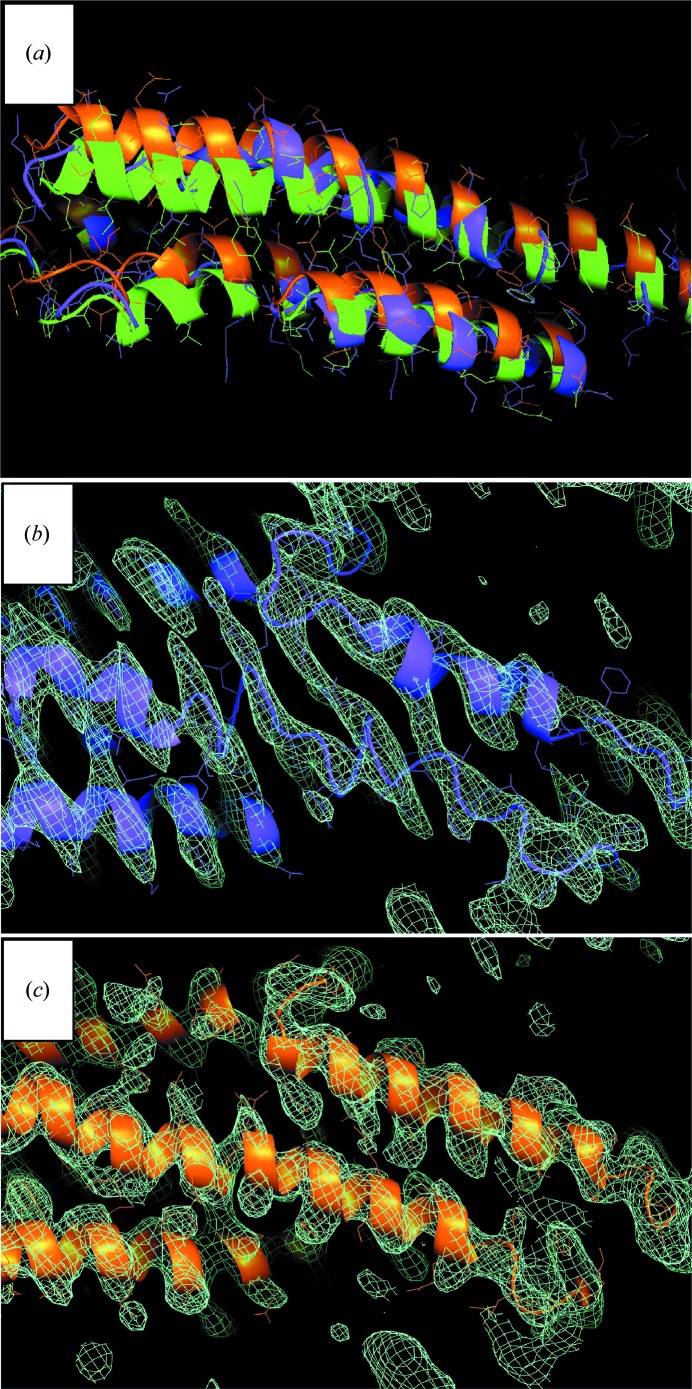
DEN refinement was essential to determine the structure of myxovirus resistance (MxA) protein (Gao *et al.*, 2011 ▶). (*a*) The starting model (green) for refinement consisted of a previously determined structure of the MxA stalk domain, a homology model of the G domain and an initial model for the BSE domain, which could be manually built into the electron-density map with phases obtained by molecular replacement. The structure obtained by DEN refinement (orange) showed better defined secondary-structure geometry compared with a structure obtained by refinement without DEN restraints (magenta). The electron density obtained from the refinement without DEN restraints (*b*) showed wrong connectivity in many places and less well defined side chains than the electron density obtained by DEN refinement (*c*).

The best combination obtained from the grid search for DEN parameters was (γ = 0.2, *w*_DEN_ = 300), yielding *R*_work_ and *R*_free_ values of 30.2 and 36.0%, respectively. All other DEN parameters used default values. The control refinement repeats without DEN restraints (w_DEN_ = 0.0) yielded *R*_work_ and *R*_free_ values of only 38.6 and 48.8%, respectively. Fig. 8 ▶(*a*) shows the starting model (green) and the models refined with (orange) and without (magenta) DEN restraints. DEN refinement maintained the helical structure and accomplished larger conformational changes than refinement without DEN (the r.m.s.d. to the starting model is 4.8 and 3.2 Å for DEN refinement and for refinement without DEN, respectively). Moreover, refinement without DEN produced severely distorted helices. The r.m.s.d. between the refined models with and without DEN restraints is 5.3 Å.

In addition to large conformational changes, DEN led to dramatic improvements of the electron-density map in the region of the stalk domain (compare Figs. 8 ▶ *b* and 8 ▶ *c* for the models refined without and with DEN restraints, respectively). Without DEN the electron density showed several wrong connections and some side chains were poorly defined (Fig. 8 ▶ *b*). Thus, manual corrections of the model would not have been possible without DEN refinement. It should be noted that the final structure deposited in the PDB (PDB entry 3szr) was re-refined against the diffraction data of a mutant MxAΔ1-32a, since the quality of that diffraction data set was improved compared with the data set used for molecular replacement and initial refinement; this final refinement yielded *R*_work_ = 26.2% and *R*_free_ = 29.5% (Gao *et al.*, 2011 ▶).

### 4.5. DEN refinement facilitates automated model building   

Here, we show that DEN refinement and automated model building work together synergistically. The ‘standard’ procedure used to determine a macromolecular X-ray crystal structure iterates over three steps: (i) computing an electron-density map with phases obtained from experiment and/or from the current model, (ii) building or rebuilding parts of the model in real space and (iii) refining the modified model in reciprocal space. For successful structure determination by such iterative model building the quality of the phases is important. Specifically, the initial phases need to be of sufficient quality in order to allow model building or correction of the initial model, as only then can a new or updated model yield better phases in the next refinement iteration. In this example, we show that DEN refinement of an initial model obtained by molecular-replacement phasing can lead to improved electron-density maps that are able to assist automated model building (Brunger, Das *et al.*, 2012 ▶).

The case considered is the crystal structure of the protein Cgl1109 (Joint Center for Structural Genomics target 376512 http://targetdb.sbkb.org/TargetDB/), a putative succinyl-diaminopimelate desuccinylase from *Corynebacterium glutamicum*. The crystal diffracted anisotropically to a resolution of 2.97 Å. In addition to the high anisotropy, the overall *B* factors were large: along the principal axes of the unit cell they are in the range 60–110 Å^2^, which made the structure determination significantly more challenging than is typically the case at this moderate resolution.

**Figure 9:**
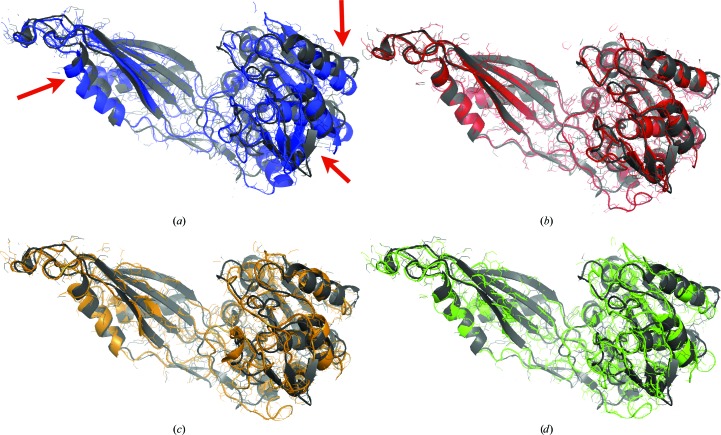
DEN refinement of Cgl1109 assisted automated model building. (*a*) The molecular-replacement solution (blue model) is shown together with the final deposited structure (gray; PDB entry 3tx8). The final refined model (gray) is further superimposed on models obtained by different refinement protocols: (*b*) DEN refinement (red), (*c*) simulated-annealing torsion-angle refinement (yellow) and (*d*) ‘standard’ refinement consisting of 200 steps of positional minimization against *E*_target_ with *w*_DEN_ = 0 (green).

The search model for molecular replacement was generated by homology modeling with *MODELLER* (Sali & Blundell, 1993 ▶) based on the template PDB entry 1vgy (chain *A*), which has a sequence identity of 28% to Cgl1109. Modeling was performed with minimal optimization using the a.very-fast() option in *MODELLER*. Using more extensive optimization with *MODELLER* did not produce a molecular-replacement solution. The minimally optimized homology model as well as the template structure itself both yielded a molecular-replacement solution with *Phaser* (McCoy *et al.*, 2007 ▶). However, this molecular-replacement model had several sequence register shifts, resulting in displacements of secondary-structural elements (red arrows in Fig. 9 ▶ *a*) when compared with the final refined model.

Manual interpretation of the electron density obtained by molecular replacement was very difficult. Therefore, the molecular-replacement solution was refined using DEN refinement with default settings, except that individual *B*-factor refinement was carried out instead of restrained grouped *B*-factor refinement, which is justified at a resolution of 3 Å. The initial model was used as the DEN reference model. The first round of DEN refinement shifted the model substantially away from the starting model and closer to the final refined model (Fig. 9 ▶ *b*, red). The corresponding *R*_free_ value was 0.444.

For comparison, simulated-annealing refinement was performed using the same protocol as used in the DEN refinement but without DEN restraints (*w*_DEN_ = 0.0). The resulting *R*_free_ value of 0.479 was significantly higher than that for the DEN-refined model. Furthermore, the secondary-structure geometry was distorted in several places (Fig. 9 ▶ *c*), resulting in poorly defined β-strands. The lower quality of this model was also reflected by a significantly lower Ramachandran score, with 47% of the residues in the favored region (as measured by *MolProbity*; Chen *et al.*, 2010 ▶) compared with 67% for the DEN-refined model. Because of serious register shifts (insertions and deletions) of the final model with respect to the starting homology model, it is difficult to compute meaningful r.m.s.d. values for the entire structure.

In addition, we tested the performance of a ‘standard’ refinement without DEN restraints; this consisted of three macrocycles of 200 steps of positional minimization and 200 steps of restrained individual *B*-factor refinement using *CNS*. Refinement was started from the molecular-replacement solution and yielded a model with an *R*_free_ value of 0.517.

After the refinement of the initial model, automatic model building was tested using the *AutoBuild* method (Terwilliger *et al.*, 2008 ▶) as implemented in *PHENIX* (Adams *et al.*, 2010 ▶). *AutoBuild* was able to build a significantly better model when starting from the DEN solution compared with when starting from the model produced by ‘standard’ refinement. The resulting *R*_cryst_ and *R*_free_ values were 0.327 and 0.418, respectively, when autobuilding from the DEN-refined model density, 0.371 and 0.457, respectively, when autobuilding from model obtained by simulated annealing with DEN, and 0.374 and 0.483, respectively, when autobuilding after ‘standard’ refinement.

The experimental phases for Cgl1109 had been determined by SeMet MAD phasing, but the initial electron density from these MAD phases was difficult to interpret. However, these phases were of benefit when used in the second round of DEN refinement using the MLHL target function. After another automatic model-building step with *AutoBuild*, the *R*_cryst_ and *R*_free_ values dropped to 0.325 and 0.372, respectively. This second round of refinement and model building resulted in only relatively small localized changes of the model, which mostly improved side-chain positions.

After these two rounds of DEN refinement and automatic model building, there were still several regions in the model that contained register shifts that could not be automatically corrected by DEN refinement and *AutoBuild* alone. Semi-automated building of these problematic regions was performed to fully refine the structure. The final refined structure of Cgl1109 (PDB entry 3tx8) yielded *R*_cryst_ and *R*_free_ values of 0.238 and 0.257, respectively. In summary, this example illustrates that DEN refinement in reciprocal space facilitates automated model building in real space, and that the combination of both methods produces better structures than with either method alone.

## 5. Outlook and concluding remarks   

The refinement of macromolecular structures at low resolution is challenging owing to the unfavorable ratio of observable data to adjustable parameters. We presented several realistic applications of DEN refinement showing that it can help with these challenges and discussing its strengths and weaknesses. The choice of these examples was intended to help the potential user devise an optimal strategy for applying DEN refinement. Over the last two years, several low-resolution crystal structures have been reported in the literature where DEN refinement was used; the number of instances where DEN refinement was used is likely to be larger since methods are often not described in detail in publications or are relegated to supplementary material that is often difficult to search.

We envision several extensions of DEN refinement that could potentially improve its performance and applicability. Instead of using only one single reference model, one could use multiple known structures, *e.g.* from different crystal structures, homology models or a combination of both. DEN restraints would then be defined from all of these reference models at the same time but each model could be weighted differently. This would increase the overall amount of information used to guide the refinement. In addition, it might be helpful to use different weights *w*_DEN_ and γ values for different regions of the reference model. In particular, if the reference model is a combination of structures of varying quality or resolution, the refinement would likely benefit if higher γ or lower *w*_DEN_ values were used for the parts of lower reliability.

Low-resolution electron density is ambiguous and more difficult to interpret. Missing density or blurred density can significantly bias the refinement and distort the model. If refinement of *B* factors is warranted considering the resolution of the crystal structure, high *B* factors may be an indicator of problematic regions. It might be helpful to couple the γ and *w*_DEN_ values used for a particular DEN restraint to the *B* factor of the two atoms that are restrained. In this way, one could use less deformable, stronger restraints for those atom pairs that are in less well defined regions of the structure.

We are currently exploring further applications of the special case of γ = 1 as discussed above. For this case we have observed that (*ab initio*) energy refinement of protein structures with molecular-dynamics simulations (*i.e.* with *E*_X-ray_ = 0 in *E*_target_) using deformable restraints with γ = 1 has the potential to improve both homology models and approximate models built from low-resolution or sparse data.

Drawing from structure-prediction methods could be powerful in extending the resolution at which automatic model building may be applied. Knowledge-based sampling, as used by structure-prediction methods, can expand the conformational space that it is feasible to explore, as well as eliminating physically impossible conformations that agree with the experimental data. Moreover, physics-based force fields may be useful to decide between alternate conformations that fit the experimental data equally well.
